# Effect of L-carnitine supplementation on lipid accumulation product and cardiovascular indices in women with overweight/obesity who have knee osteoarthritis: a randomized controlled trial

**DOI:** 10.1186/s41927-022-00286-8

**Published:** 2022-09-22

**Authors:** Abbas Ali Sangouni, Farnaz Baghban, Maryam Khosravi, Hassan Mozaffari-Khosravi, Ali Dehghan, Mahdieh Hosseinzadeh

**Affiliations:** 1 Nutrition and Food Security Research Center, School of Public Health, Shahid Sadoughi University of Medical Sciences, Yazd, Iran; 2 Department of Nutrition, School of Public Health, Shahid Sadoughi University of Medical Sciences, Yazd, Iran; 3 Department of Nutrition, Faculty of Medicine, Mashhad University of Medical Sciences, Mashhad, Iran; 4 Department of Internal Medicine, Shahid Sadoughi Hospital, Shahid Sadoughi University of Medical Sciences, Yazd, Iran

**Keywords:** Knee osteoarthritis, L-carnitine, Lipid accumulation product, Cardiovascular risk

## Abstract

**Background:**

Osteoarthritis is associated with obesity, dyslipidemia and cardiovascular diseases. It has been hypothesized that L-carnitine can improve cardiovascular risk factors. We aimed to investigate the effect of L-carnitine supplementation on lipid accumulation product (LAP) and atherogenic indices in women with overweight/obesity who have knee osteoarthritis.

**Methods:**

In this double-blind randomized controlled trial, seventy-six women with overweight/obesity who had knee osteoarthritis were assigned into the intervention group and control group for 12 weeks. The intervention group received 1000 mg/day L-carnitine as capsule, and the control group received placebo. The primary outcomes were LAP, atherogenic index of plasma (AIP), atherogenic coefficient (AC) and Castelli risk index II (CRI-II).

**Results:**

We found no significant difference between the groups in baseline values of LAP, AIP, AC and CRI-II. After the intervention, a significant reduction in LAP was observed in intervention group compared to the control group (− 11.05 (− 28.24 to 0.40) vs. − 5.82 (− 24.44 to 2.68); P = 0.03). However, there was no significant difference between two groups in AIP (− 0.05 ± 0.16 vs. − 0.01 ± 0.13; P = 0.19), AC (− 0.40 ± 0.81 vs. − 0.30 ± 0.67; P = 0.67) and CRI-II (− 0.20 ± 0.76 vs. − 0.21 ± 0.47; P = 0.11).

**Conclusions:**

L-carnitine supplementation for 12 weeks can improve LAP, but it has no effect on cardiovascular outcomes. To reach a definitive conclusion, further clinical trials with larger sample sizes and higher dosages of L-carnitine are needed.

***Trial registration*:**

Registered on 27/4/2017 at Iranian Registry of Clinical Trials IRCT2017011932026N2.

## Background

The prevalence of osteoarthritis is increasing and has become a serious healthcare problem [1, 2]. Patients with osteoarthritis are suffering from pain, stiffness, deformity, and loss of functional capacity [1, 3, 4]. Knee osteoarthritis is a chronic musculoskeletal disease [3, 5]. Obesity, gender (female), advanced age and genetic contributed to the development of knee osteoarthritis [2–5]. Oxidative stress, obesity and dyslipidemia are involved in the pathogenesis of osteoarthritis [6–8]. On the other hand, the prevalence of cardiovascular disease (CVD) risk factors such as dyslipidemia, obesity and hypertension is higher in individuals with osteoarthritis compared to the subjects without osteoarthritis [9]. Atherogenic indices such as atherogenic index of plasma (AIP), atherogenic coefficient (AC) and Castelli risk index II (CRI-II) are used to estimate the cardiovascular [10, 11]. The equations of these indices are based on lipid profile [11]. It has been suggested that atherogenic indices can predict cardiovascular risk better than low density lipoprotein-cholesterol (LDL-c), triglyceride (TG) or total cholesterol (TC) alone [10, 11]. In addition, lipid accumulation product (LAP) as a novel and available index can predict visceral obesity and cardiometabolic risk [12–15]. The LAP is based on TG and waist circumference (WC) [13].

L-carnitine (4-N-trimethylammonium-3-hydroxybutyric acid) is synthesized from amino acids lysine and methionine [16]. L-carnitine regulates the metabolism of fatty acids as well as ATP synthesis [16–18]. The dietary sources of L-carnitine are meat, fishes and dairy products [16, 17]. Some studies investigated the effect of L-carnitine in obesity, insulin resistance and oxidative stress [19–23]. The recent evidence suggested the beneficial effects of L-carnitine on risk factors of knee osteoarthritis such as obesity and dyslipidemia [20, 21, 23–25]. The studies that evaluated the effect of L-carnitine in patients with osteoarthritis are scarce [19, 26, 27]. In addition, only one study examined the effect of L-carnitine supplementation on LAP and atherogenic indices in women with polycystic ovary syndrome (PCOS) [28]. Therefore, we designed a clinical trial to investigate the effect of L-carnitine supplementation on LAP and cardiovascular indices in women with overweight/obesity who have knee osteoarthritis.

## Methods

### Recruitment and eligibility screening

A total of one hundred women with knee osteoarthritis were screened at the Khatam Al-Anbia clinic of Rheumatology Department affiliated with Shahid Sadoughi University of Medical Sciences, Yazd, Iran. The inclusion criteria were as follows: subjects with knee osteoarthritis based on clinical criteria, aged more than 45 years, and body mass index (BMI) between 25–35 kg/m^2^. The exclusion criteria were as follows: former or planned knee-joint replacement, other rheumatic diseases, severe liver or kidney diseases, thyroid diseases, severe heart diseases, pregnancy, taking pharmacological treatments for obesity, lipid lowering medications and nonsteroidal anti-inflammatory drug (NSAID), taking multivitamin, minerals, or other nutritional supplements. Unwillingness to continue the trial, and poor compliance (consuming L-carnitine and placebo less than 80% of the expected amount) were considered as drop-out criteria.

### Trial design

We conducted a 12-week double-blind randomized controlled trial (RCT) from September 2018 to December 2018. The study protocol was confirmed by the ethical committee of Shahid Sadoughi University of Medical Sciences and Health Services in Yazd, Iran (IR.SSU.SPH.REC.1395.45). In addition, the protocol was registered at the Iranian clinical trials website on 27/4/2017 (IRCT2017011932026N2) with URL: https://www.irct.ir/trial/25050. A trained person assigned the subjects into the intervention and control groups through simple randomization using random number table (random allocation software) [29]. Allocation concealment was conducted using opaque sealed envelopes to prevent selection bias by concealing the allocation sequence from those assigning participants to the intervention groups. Subjects and investigators were blinded to the intervention assignment.

### Intervention

The intervention group received 1000 mg/d L-carnitine as capsule and the control group received the same amount of placebo (cellulose). Every 2 weeks, the subjects received capsules. To estimate the compliance rate, the empty sachets of capsules were collected at the end of each month. The capsules of L-carnitine and placebo had similar appearance. All capsules were produced by Karen Pharmaceuticals Co., Yazd, Iran. In addition, all subjects followed a low-calorie diet. A registered dietitian estimated the energy expenditure of each patient through Harris-Benedict formula [30]. The composition of the diet was as follows: 50% to 60% carbohydrates, 15% to 20% proteins, and less than 30% total fat.

### Dietary intake and physical activity assessments

Energy and macronutrients intakes were assessed by a 3-day (1 weekend day and 2 nonconsecutive weekdays) 24-h recall questionnaire at the baseline and week 12. Analysis of the dietary intakes was performed using a Nutritionist IV software (Warrington, United Kingdom).

In addition, physical activity was assessed at the baseline and after intervention using a long form of International Physical Activity Questionnaire (IPAQ) [31].

### Laboratory and anthropometric evaluations

Laboratory and anthropometric assessments were performed at the baseline and after intervention. To measure the serum concentrations of TC, TG and high density lipoprotein-cholesterol (HDL-c), 5 ml blood was collected after 8 h fasting. The samples were centrifuged and immediately frozen and stored at − 70 °C. Using an autoanalyzer (AVIDA 1800 chemistry system; Siemens, United Kingdom) and Pars Azmoon kits, the serum concentrations of TC, TG and HDL-c were measured. In addition, estimating LDL-c was performed by the Friedewald’s equation [32].

Anthropometric measurements were performed at the baseline and after intervention. The weight was measured using a portable digital scale (Omeron BF511, Japan) with an accuracy of 100 g. Assessment of height was performed utilizing a stadiometer with an accuracy of 0.1 cm. Measuring WC was performed under the standard protocol by a measuring tape. We calculated body mass index (BMI) using the following formula: weight (kg)/height squared (m^2^).

### Indices

Calculating LAP [13], AIP [10], AC [33] and CRI-II [34] was performed at baseline and after intervention using the following equations:

- LAP_women_ = (WC − 58) × TG [13].
- AIP = log (TG/HDL-c) [10].
- AC = (TC-HDL-c)/HDL-c [33].
- CRI-II = LDL-c/HDL-c [34].

### Sample size and statistical analysis

Our previous article reported the results of clinical symptoms, lipid profile, C-reactive protein and malondialdehyde [26], and the optimal sample size was estimated to be 38 for each group, by assuming confidence interval, α = 0.05, and power = 80%. In addition, power analysis was performed and power = 80% was obtained for AC. To analyze the data, a statistical package for social science (SPSS) software (Chicago, Illinois, USA) version 24 was used. An intention-to-treat (ITT) approach using last observation carried forward (LOCF) analysis method was carried out. In this method, a missing follow-up visit value is replaced by the last observed value. ITT analysis includes every subject who is randomized according to randomized treatment assignment. It ignores noncompliance, protocol deviations, withdrawal, and anything that happens after randomization. Assessment of normal distributions was performed by Kolmogorov–Smirnov test. We used an independent t-test (for parametric variables), and Mann–Whitney U test (for non-parametric variables) to compare the variables between groups. In addition, analyzing parametric and non-parametric data within each group was performed using paired t-test and Wilcoxon signed rank test, respectively. To identify the differences between groups after adjusting for covariates, univariate analysis of covariance (ANCOVA) was carried out. P ≤ 0.05 was significant.

## Results

### Characteristics and anthropometric variables

**Fig. 1:**
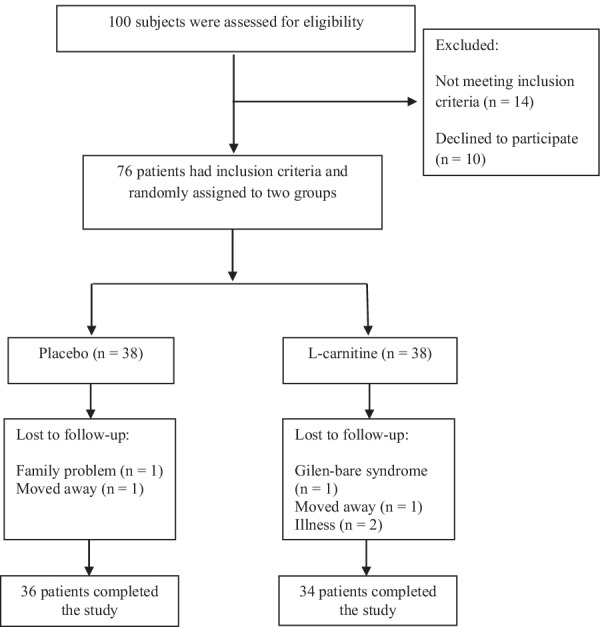
Flowchart of participant eligibility, screening, and follow-up

Six participants were excluded from the trial due to lost to follow-up. Finally, a total of 70 participants completed the trial (Fig. 1). The percentage of adherence to the study protocol was 92.1% (89.4% in intervention group and 94.7% in placebo group). We found no significant difference between groups in baseline parameters including age, education, occupational status, physical activity, energy intake, macronutrients intake, anthropometric variables and lipid profile (P > 0.05) (Table 1). After intervention, there was no difference between groups in weight, BMI, WC and energy intake (P > 0.05) (Table 2). In addition, no serious adverse effect was reported during the trial.

**Table 1:** Baseline characteristics in women with knee osteoarthritis

	L-carnitine (n = 38)	Placebo (n = 38)	P
Age (y)	55.01 ± 7.12	54.43 ± 7.80	0.73
*Education*			0.29
Illiterate	3 (7.9)	1 (2.6)	
Elementary school graduate	18 (47.4)	20 (52.6)	
Middle/high school graduate	13 (34.2)	14 (36.8)	
University graduate	4 (10.5)	3 (7.9)	
*Occupational status*			0.42
Housewife	30 (78.9)	34 (89.4)	
Employee	3 (13.9)	2 (5.3)	
Retired	5 (7.2)	2 (5.3)	
Physical activity	548.01 ± 113.4	540.01 ± 62.25	0.43
Energy (kcal)	1426.21 ± 344.91	1377.72 ± 406.44	0.57
Carbohydrate (g)	174.76 ± 37.21	177.38 ± 66.75	0.83
Fat (g)	60.83 ± 24.77	56.19 ± 27.55	0.44
Protein (g)	50.52 ± 19.342	50.63 ± 22.172	0.45
TC (mg/dl)	226.74 ± 50.55	220.71 ± 42.70	0.57
TG (mg/dl)	185.53 ± 92.50	193.63 ± 80.05	0.68
HDL-c (mg/dl)	64.97 ± 26.35	64.08 ± 19.60	0.86
LDL-c (mg/dl)	136.13 ± 43.71	126.92 ± 35.33	0.31

P values are computed by independent t-test and data are expressed as mean ± standard deviation (SD), while for Education and Occupational status, P values are computed by chi-square test and data are expressed as number (%)

MET-h: metabolic equivalent task hours; BMI: body mass index; WC: waist circumference; TC: total cholesterol; TG: triglyceride; HDL-c: high density lipoprotein-cholesterol; LDL-c: low density lipoprotein-cholesterol

**Table 2:** Anthropometric parameters and energy intake in women with knee osteoarthritis

Variables	L-carnitine (n = 38)	Placebo (n = 38)	*P*^†^
*Weight, kg*			
Baseline	78.69 ± 10.86	78.90 ± 12.17	0.93
Week 12	76.22 ± 11.06	77.05 ± 12.48	0.75
*P*	< 0.001	< 0.001	
Mean difference	− 2.47 ± 1.81	− 1.85 ± 1.74	0.13
*BMI, kg/m*^*2*^			
Baseline	33.03 ± 6.67	32.10 ± 4.29	0.47
Week 12	31.95 ± 6.32	31.35 ± 4.39	0.63
*P*	< 0.001	< 0.001	
Mean difference	− 1.08 ± 0.87	− 0.75 ± 0.7	0.07
*WC, cm*			
Baseline	105.13 ± 9.04	105.92 ± 10.57	0.72
Week 12	99.81 ± 11.17	102.47 ± 10.32	0.28
*P*	< 0.001	< 0.001	
Mean difference	− 5.32 ± 5.60	− 3.45 ± 3.38	0.08
*Energy intake, Kcal*			
Baseline	1426.21 ± 344.91	1377.72 ± 406.44	0.57
Week 12	1325.18 ± 154.48	1333.88 ± 153.92	0.80
*P*	0.11	0.52	
Mean difference	− 101.03 ± 388.86	− 43.84 ± 417.09	0.53

P values are computed by independent t-test and data are expressed as mean ± standard deviation (SD)

*P*: resulted from within each group comparison

*P*^†^: resulted from comparing the means of each variable at the end of the study, between groups

BMI: body mass index; WC: waist circumference

### Outcomes

There was no significant difference between groups in terms of LAP (P = 0.28), AIP (P = 0.93), AC (P = 0.11) and CRI-II (P = 0.15) at the baseline (Table 3). At the end of the intervention, a significant difference was found between groups in terms of LAP (P = 0.05) and CRI-II (P = 0.03); however, there was no significant difference between groups in AIP (P = 0.37) and AC (P = 0.22) (Table 3).

**Table 3:** Effect of L-carnitine on indices in women with knee osteoarthritis*

Indices	L-carnitine (n = 38)	Placebo (n = 38)	*P*^†^	*P*^††^
*LAP*				
Baseline	88.76 (69.02 to 105.40)	100.57 (70.05 to 138.81)	0.28*	0.03
Week 12	74.70 (44.24 to 104.92)	101.69 (61.25 to 113.22)	0.05*	
*P*	0.002**	0.05**		
Mean change of LAP	− 11.05 (− 28.24 to 0.40)	− 5.82 (− 24.44 to 2.68)	0.28*	
*AIP*				
Baseline	0.09 ± 0.19	0.09 ± 0.19	0.93	0.19
Week 12	0.04 ± 0.17	0.08 ± 0.20	0.37	
*P*	0.04	0.57		
Mean change of AIP	− 0.05 ± 0.16	− 0.01 ± 0.13	0.21	
*AC*				
Baseline	2.83 ± 0.79	2.53 ± 0.83	0.11	0.67
Week 12	2.43 ± 0.60	2.23 ± 0.81	0.22	
*P*	0.004	0.008		
Mean change of AC	− 0.40 ± 0.81	− 0.30 ± 0.67	0.57	
*CRI-II*				
Baseline	2.32 ± 0.77	2.07 ± 0.73	0.15	0.11
Week 12	2.12 ± 0.47	1.85 ± 0.57	0.03	
*P*	0.11	0.01		
Mean change of CRI-II	− 0.20 ± 0.76	− 0.21 ± 0.47	0.94	

Values for AIP, AC and Castelli risk index II are presented as mean ± standard deviation (SD), while for LAP are presented as median and quartile range

*P values are computed by Mann–Whitney U test

**P values are computed by Wilcoxon test

*P*: resulted from within each group comparison

*P*^†^: resulted from comparing the means of each variable at the end of the study, between groups

*P*^††^: adjusted based on education and baseline values of parameters using univariate ANCOVA

LAP: lipid accumulation product; AIP: atherogenic index of plasma; AC: atherogenic coefficient; CRI-II: Castelli risk index II

A significant reduction in LAP was observed in the L-carnitine group compared to the placebo group (− 11.05 (− 28.24 to 0.40) vs. − 5.82 (− 24.44 to 2.68); P = 0.03). However, there was no significant difference between groups in AIP (mean changes: − 0.05 ± 0.16 vs. − 0.01 ± 0.13; P = 0.19), AC (mean changes: − 0.40 ± 0.81 vs. − 0.30 ± 0.67; P = 0.67) and CRI-II (mean changes: − 0.20 ± 0.76 vs. − 0.21 ± 0.47; P = 0.11) (Table 3).

## Discussion

To the best of our knowledge, the present study is the first RCT that examined the effect of L-carnitine on LAP, AIP, AC and CRI-II in women with knee osteoarthritis. We demonstrated that L-carnitine supplementation for 12 weeks can reduce LAP. However, L-carnitine supplementation had no impact on AIP, AC and CRI-II.

The LAP index is known as an available and accurate scoring system to estimate cardiometabolic risk and visceral obesity [12–15]. A RCT evaluated the effect of 12-week L-carnitine supplementation (1000 mg/day) on LAP, and reported that L-carnitine has no effect on LAP among women with PCOS [28]. The crude results of our study was similar to the study of Sangouni et al. [28]; however, we found a significant reduction of LAP after adjusting for education of participants. WC and TG are used in the equation of LAP [13]. The study of Malaguarnera et al. [22] demonstrated that 2000 mg/day L-carnitine supplementation can improve the levels of TG in patients with T2DM. In addition, Lee et al. [35] found a slight reduction in TG after 12-week L-carnitine supplementation in patients coronary artery disease. However, the study of Malek Mahdavi et al. [19] reported that 750 mg/day L-carnitine has no effect on TG in women with osteoarthritis. In addition, Samimi et al. [23] conducted a study among women with PCOS, and showed that 250 mg/day L-carnitine supplementation for 12 weeks has no effect on TG. It seems, higher dosages of L-carnitine can improve the levels of TG. On the other hand, a RCT found a significant reduction of WC in patients with PCOS who received L-carnitine supplementation for 12 weeks [36]. However, the RCT of Samimi et al. [23] reported that L-carnitine supplementation (250 mg/day) for 12 weeks has no effect on WC in women with PCOS. Probably, high dosage of L-carnitine supplementation has a beneficial effect on body composition. A meta-analysis of RCTs revealed that L-carnitine supplementation between 1800 and 4000 mg/day can improve some anthropometric variables [37]. However, further RCTs with larger sample sizes are needed to determine the long-term effects of L-carnitine supplementation and subsequently reach a reliable conclusion. It has been suggested that L-carnitine can reduce lipid accumulation through inducing lipid beta-oxidation, regulating hormone-sensitive lipase, acyl-coenzyme A oxidase and peroxisome proliferator-activated receptor-gamma (the main factors involved in the lipid catabolism or adipogenesis), regulating appetite control, decreasing energy intake and increasing energy expenditure [20, 21].

We demonstrated that AIP, AC and CRI-II did not change after 12-week L-carnitine supplementation. Consistent with our findings, a RCT showed that L-carnitine supplementation (1000 mg/day) for 12 weeks could not improve AIP, AC and CRI-II in patients with PCOS [28]. AIP, AC and CRI-II are based on lipid profile [10, 33, 34]. The study of Baghban et al. [26] demonstrated that L-carnitine supplementation (1000 mg/day) has no significant effect on TG, TC, LDL-c and HDL-c in women with knee osteoarthritis. In addition, the RCT of Samimi et al. [23] reported that 250 mg/day L-carnitine supplementation for 12 weeks could not improve TG, TC, LDL-c and HDL-c in women with PCOS. Moreover, a RCT found that L-carnitine supplementation for 12 weeks with dose of 1000 mg/day has no impact on lipid profile in patients with PCOS [38]. However, the study of Malaguarnera et al. [22] demonstrated a significant reduction of TG, LDL-c and oxidized LDL-c in patients with T2DM who received 2000 mg/day L-carnitine supplementation for 12 weeks. According to evidence, the main reason of some inconsistencies in this field can be the difference in dosage of L-carnitine. It seems, high dosage of L-carnitine has some beneficial effect on lipid profile. A meta-analysis reported that L-carnitine intake more than 2000 mg/day can improve the blood lipid parameters [39]. High dose of L-carnitine can stimulate apolipoprotein-A1 production, decrease synthesis of TG and esterification toward acetylcarnitines formation, increase lipid beta-oxidation, regulate hormone-sensitive lipase and induce lipid catabolism [25, 40, 41].

We declare that our research group reported the findings of clinical symptoms, anthropometric parameters, lipid profile, C-reactive protein and malondialdehyde [26], and we used the same data for the present article. To follow the principals of ethics in research, we clarify that Fig. 1 and some important information of the mentioned article [26] were added to the present article.

As an important strength, the present study was the first RCT that investigated the effect of L-carnitine supplementation on indices such as LAP, AIP, AC and CRI-II in women with knee osteoarthritis. However, the present study had some important limitations. The dosage of L-carnitine that we used for this study was low. In addition, the sample size of this study was small. A small sample size may make it difficult to reach a firm conclusion.

## Conclusions

In conclusion, L-carnitine supplementation (1000 mg/day) for 12 weeks improved LAP, but it has no effect on atherogenic indices. Studies with larger sample sizes as well as higher dosages of L-carnitine are required to demonstrate the real efficacy of L-carnitine.

## Data Availability

The datasets generated and analyzed during the current study are not publicly available due to their containing information that could compromise the privacy of research participants, but are available from the corresponding author, Mahdieh Hosseinzadeh, on reasonable request.

## Abbreviations

ANCOVA: Analysis of covariance
CVD: Cardiovascular disease
HDL-c: High density lipoprotein-cholesterol
IPAQ: International physical activity questionnaire
ITT: Intention-to-treat
LAP: Lipid accumulation product
LDL-c: Low density lipoprotein-cholesterol
LOCF: Last observation carried forward
NAFLD: Non-alcoholic fatty liver disease
PCOS: Polycystic ovary syndrome
RCT: Randomized controlled trial
SPSS: Statistical package for social science
T2DM: Type 2 diabetes mellitus
TC: Total cholesterol
TG: Triglyceride
VAI: Visceral adiposity index
WC: Waist circumference
